# Anticancer action of plant products: changing stereotyped attitudes

**DOI:** 10.37349/etat.2022.00092

**Published:** 2022-08-15

**Authors:** Katrin Sak

**Affiliations:** NGO Praeventio, 50407 Tartu, Estonia; Istituto Nazionale Tumori “Fondazione Pascale” Via Mariano Semmola, Italy

**Keywords:** Natural anticancer products, phytochemicals, chemotherapeutic drugs

## Abstract

Compared to humans, plants can synthesize an extremely diverse array of chemical compounds, including phenolic acids, flavonoids, stilbenes, lignans, terpenoids, alkaloids, and many other types of secondary metabolites that have been demonstrated to exert important bioactivities and impacts on the human health. As a result of extensive and sustained efforts, some phytochemicals like vincristine, vinblastine, and paclitaxel have already been approved as anticancer drugs today, while several others are under clinical trials. However, despite this remarkable success, studies on anticancer action of plant-derived products have been and paradoxically are still in some places, mixed up with alternative approaches and thereby considered non-credible, especially in regions where the role of traditional medicine has not been historically so prevalent as in several Asian countries. As a result, only about 10% of higher plants have been explored regarding the potential therapeutic effects of their constituents. Moreover, as one function of secondary metabolites includes the protection of plants against diverse environmental stresses, the content and composition of these phytochemicals might importantly vary between different regional habitats. Therefore, the stereotyped attitudes to plant products as something related to alternative medicine must be changed to identify new lead molecules for novel anticancer drugs. It is possible that plants still harbor an important spectrum of pharmaceutically interesting, but still unidentified, chemical compounds.

Although plants have been used as remedies against malignant disorders in traditional medicine already for centuries, a systematic approach to the identification of novel anticancer agents from plant-derived products began only with launching the screening program of the United States National Cancer Institute (NCI) in 1960 [1–3]. Within this large-scale program, during the following twenty years (1960–1982), extracts of almost 3,400 plant species were explored regarding their possible anticancer potential towards a panel of rodent and human tumor systems [2, 4]. As a result, efficient anticancer agents vinblastine and vincristine were isolated from the Madagascar periwinkle *Catharanthus roseus* (L.) G.Don, podophyllotoxin from the roots of mayapple *Podophyllum peltatum* Linnaeus, paclitaxel from the bark of Pacific yew *Taxus brevifolia* Nutt., and camptothecin from the bark of happy tree *Camptotheca acuminata* Decne. [2, 3]. Both the alkaloids vinblastine and vincristine, terpenoid paclitaxel, as well as semisynthetic derivatives of podophyllotoxin (etoposide, teniposide) and camptothecin (topotecan, irinotecan) are nowadays extensively used in the treatment of various malignancies in the modern clinical practice [2, 3]. Moreover, despite the expansion of combinatorial chemistry in the 1990s, and the development of targeted and immune therapies, these “old” drugs have remained valuable cornerstones for cancer treatment still to date, continually presenting a subject for both mechanistic as well as combinatorial studies [3]. This means that no modern medications have been able to discard the originally plant-derived chemotherapeutic drugs and their next-generation semisynthetic derivatives from clinical use; in other words, time has only justified the plant-based approach for identifying anticancer agents, proving in turn the enormous capacity of plants to biosynthesize novel chemical entities with important health effects.

In general, plants are producing a wide variety of compounds to protect themselves against diverse environmental stresses, such as ultraviolet (UV) radiation and numerous microbial pathogens and herbivores [5–7]. Therefore, it is not surprising that many phytochemicals have exhibited potent activities also against human diseases, including various forms of malignancies [3]. However, up to now, only about 10% of such natural abundance, consisting of over 250,000 higher plant species, have been investigated concerning their possible therapeutic potential against different types of diseases [8–10]. Accordingly, the great majority of the plant kingdom is still unexplored towards the chemical composition and bioactivities of constituents, possibly harboring a plethora of unidentified structures and hopefully also some new therapeutic strategies.

There are several reasons behind the limited progress in the investigation into natural anticancer compounds. Firstly, these studies are very laborious, requiring knowledge about different disciplines, including physical, analytical, and bioorganic chemistry, but also access to specific technological infrastructure for the identification of novel chemical entities and screening of their potential bioactivities. In this way, the costs of such studies cannot be underestimated. Extracting plant components is always a very complicated and challenging task, associated with the choice of proper solvents as well as the potential risks of contamination and decomposition, which must all be carefully considered [11]. Secondly, and probably even more importantly, the attitude of some scientific communities to natural compounds as “alternative medications” has substantially impeded the development of research on the biosynthetic factories of plants, setting pharmacological studies on phytochemicals in strict contrast to evidence-based western medicine. However, without launching the plant screening program by the NCI in 1960 and investigating into pharmacological properties of plant bioactive constituents, such well-known anticancer drugs like paclitaxel and vincristine would probably have never been discovered and reached everyday clinical practice, just because of their very complex chemical structures that are impractical for total synthesis [3]. Furthermore, due to cultural and religious beliefs and values, the interest in studies of natural anticancer compounds has also been largely regional. In Figure 1, the numbers of PubMed database-indexed articles containing the keywords “natural” and “anticancer” are compared by different countries (data presented as of June 13th, 2022). It is evident that in the regions with a very strong and centuries-old background of traditional medicine, including Asian countries like China, India, Japan, Iran, and Saudi Arabia, research into the constituents and bioactivities of medicinal plants is highly popular and also productive. The academic interest in the investigation into phytochemicals as potential drug candidates against malignant disorders has been very high also in the USA; probably related, at least in part, to the national financial support and collaborative relations provided to such research, like the exploratory screening programs of the NCI. On the other hand, the interest in studies on natural anticancer compounds has been very limited in several Nordic and also Baltic countries. It is important to mention that this field does probably not belong also to the most essential topics of national research and innovation strategies in these regions. At the same time, it is well accepted that not only the content of specific phytochemicals in plants but also their composition and structural diversity can be essentially dependent on environmental factors, including exposure to light, temperature, soil water, salinity, and fertility [12, 13]. Therefore, the production, accumulation, and release of plant secondary metabolites (terpenes, phenolics, nitrogen-containing compounds, and organosulfur compounds) might be altered by different abiotic stresses [12, 13]. As these compounds play an important role in the protection of plants against harmful circumstances, their biosynthesis is commonly intensified in harsh conditions often characteristic of northern regions [12, 13]. Moreover, some agents can be synthesized only in certain circumstances [12, 13]. For example, the production of phenolic compounds like anthocyanins, and probably also some other types of flavonoids, is generally increased in plants growing in the northern latitudes with typical long cool days and cold nights as compared to the same species in the south [13]. As flavonoids are known also as plant pigments, such climate-based regulation of their synthesis might explain also the hastened coloration of berries of grape plants *Vitis vinifera* L. grown under lower night temperatures [14] or darker flowers of *Plantago lanceolata* L. cultivated at cooler ambient temperatures [15]. Accordingly, plants growing in northern climate conditions can produce some unidentified compounds that could probably not be present in their counterparts from southern or eastern regions. As these phytochemicals might possess a number of important bioactivities, presenting potential lead molecules for drug discovery processes, the research on plant-derived anticancer agents should be intensified, equally all over the world. It is highly possible that plant biosynthetic factories harbor still a huge potential for pharmaceutical and biomedical applications.

**Figure 1.**
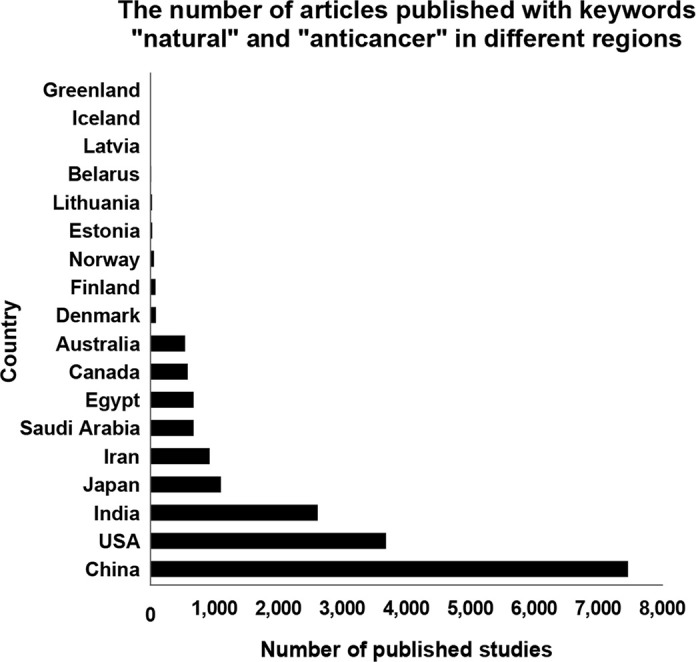
The number of studies published in different countries and indexed in the PubMed database with the keywords “natural” and “anticancer” (as of June 13th, 2022)

The question remains how to encourage the studies on natural anticancer products in countries where such research field is not a reasoned part of a common cultural or religious area (like in Chinese medicine) and the attitude to this subject is rather a sceptical than enthusiastic? What to do when a plant- derived compound becomes attractive only after being packaged into a drug bottle but not when it is biosynthesized in plants? How a bioactive phytochemical can reach the drug bottle at all when this research is disregarded? Therefore, do we need to start some new plant screening programs for identifying novel lead compounds for anticancer agents, especially in northern regions? Considering the rapidly increasing incidence rate of new cancer cases all over the world with a 47% rise expected from 2020 to 2040, reaching 28.4 million new cancer cases in 2040 [16], the fight against malignant disorders definitely needs common intensified efforts over the coming years.

## Abbreviations

NCI: National Cancer Institute

## Declarations

### Copyright

© The Author(s) 2022.
